# Clinical and translational science award hubs in learning health systems: development of the engine-drivetrain model

**DOI:** 10.1186/s12967-026-08127-9

**Published:** 2026-04-14

**Authors:** Octavian C. Ioachimescu

**Affiliations:** 1 Department of Medicine, Division of Pulmonary, Critical Care and Sleep Medicine, Medical College of Wisconsin, Milwaukee, Wisconsin USA; 2 Clement J. Zablocki Veteran Affairs medical Center, Milwaukee, Wisconsin USA

## Abstract

**Background:**

Over the past two decades, biomedical research has made extraordinary progress, at amazing speeds and with astounding computational capabilities. Even in very agile organizations, these fast-paced advances represent significant barriers to nimble and successful implementation, scaling, integration, cultural shaping, and ongoing education. Learning at individual, team, organization or system levels is paramount to the good functioning of all health systems.

**Main Body:**

Academic health institutions are uniquely positioned to drive innovation, improve patient outcomes, and cultivate future leaders by integrating well-managed clinical operations, breakthrough research, and outstanding education. The Learning Health System (LHS) framework provides a powerful model of integration, leveraging iterative cycles of discovery, learning and improvement by employing robust operational management, analytical capabilities, implementation and dissemination capacity and functionalities. Central to this model are three interconnected phases: Practice to Data, Data to Knowledge, and Knowledge to Practice, which form the ‘implementation arc’ or the LHS cycle. At each junction between these phases, the incorporation of external evidence and the active engagement of community members or stakeholders serve as critical pillars, ensuring that learning is both rigorous and relevant.

**Conclusions:**

First, we offer a modified design and conceptualization of LHS as a four-cycle, mission-based model centered by its community, and strengthened by external evidence and data as distinct inputs, at multiple points of the cycle. Second, we propose that the Clinical and Translational Science Award (CTSA) hubs’ structures and functions can represent the strong operational engine of the LHS plant, while community represents the central axle and transmission of movement in the central mechanism of the LHS. As such, the CTSA hubs can serve as a robust machinery for the clinical, research, and educational cycles in learning health systems.

**Randomized control trial number:**

Not applicable.

## Key Messages

What is Known

What is the Gap

What is Novel Here

• Learning is an activity that encapsulates systematic data collection, thorough analysis to create useful insights, and robust feedback loops that incorporate those insights into the daily praxis or activities for improved outcomes.

• A Learning Health System (LHS) is a complex ecosystem in which science, informatics, incentives, and culture are aligned for innovation and continuous improvement, with best practices seamlessly embedded in the delivery processes.

• Clinical and Translational Science Award (CTSA) hubs have complex structures, cores and functions, and aim to facilitate learning and continuous improvement at individual, institutional, and system levels.

What roles can a CTSA hub have in designing, building and consolidating a LHS?

• We offer a modified design and conceptualization of LHS as a four-cycle, mission-based model centered by its community, and strengthened by external evidence and data as distinct inputs, at multiple points of the cycle.

• We propose that the CTSA hubs become strong operational *engines* of learning that operate simultaneously at all levels by integrating clinical practice, education, research, and governance activities.

• In this model, the community represents the *main axle and the variable transmission* of movement in the central mechanism of future LHS plants.

## Prelude (Allegro): Introduction

Learning is a complex activity that encapsulates systematic internal and external data collection, thorough analysis to create useful insights, and healthy feedback loops that incorporate the insights into the daily practice or usual activities for improved outcomes. A Learning Health System (LHS) is a complex ecosystem in which science, informatics, incentives, and culture are aligned for innovation and continuous improvement, with best practices seamlessly embedded in the delivery processes [1]. LHS deals with new knowledge generation, collection and analysis, implementation, dissemination and adaptation via finely tuned processes. LHS bring together in carefully orchestrated fashion human operators, scientific discoveries, innovation, technology, informatics, and clinical practice. LHS aims to integrate and optimize patient care, research, and education, with all stakeholders actively engaged in the evidence-based continuum of practice.

In 2011, National Institutes of Health (NIH) created the National Center for Advancing Translational Science (NCATS) in order to pursue, encourage, catalyze and grow funding opportunities for *disruptive translational innovation* via intramural and extramural mechanisms [2]. Over the next decades, the NCATS became the funding agency for more than 60 Clinical and Translational Science Award (CTSA) hubs with complex structures, cores, and functions that aim to facilitate learning and continuous improvement at individual, institutional, and system levels. This year marks two decades of CTSA hub activity in the United States of America.

We offer here first a modified design and conceptualization of LHS as a 4-cycle (or wheel), mission-based model centered by its community, and strengthened by external evidence and data as distinct inputs, at multiple points of the cycle. Second, we propose that the CTSA hubs’ structures and functions can represent the strong operational engine [3, 4], while community represents the central axle and the variable transmission of motion in the central mechanism of the LHS plant (the LHS-CTSA engine-drivetrain model).

### First movement (Andante): background - traditional LHS framework

**Fig. 1:**
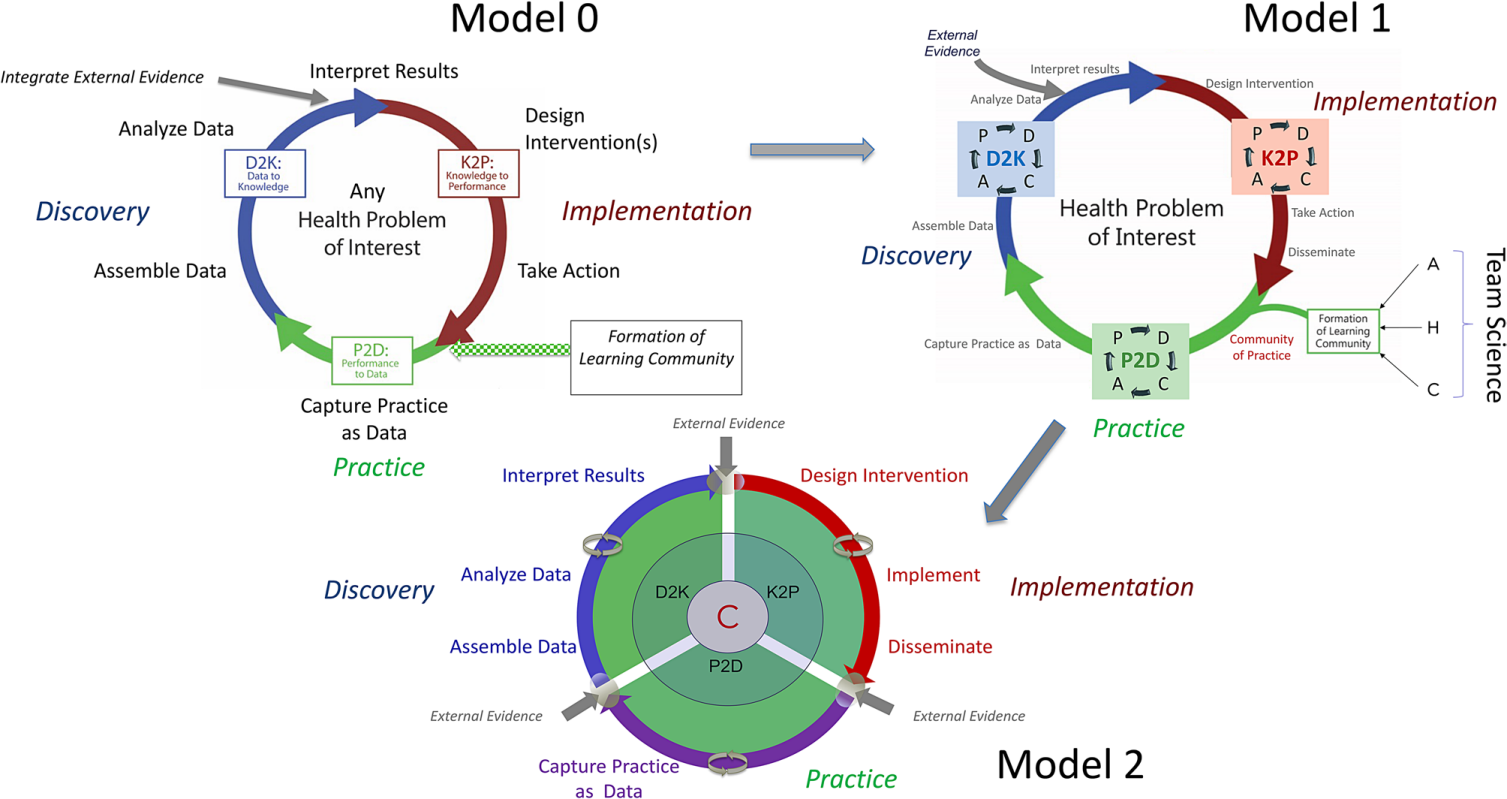
In the **LHS Model 0** (adapted from Friedman C.P.), the starting point is represented by the formation of a learning community, while external data or new discoveries are incorporated in the Discovery or Data Analysis segment of the LHS cycle or arc. In the **LHS Model 1** (modified from Friedman C.P.), PDCA (Plan-Do-Check-Act) improvement cycles are incorporated in each LHS segment, while the learning/practice community is established on a trilateral mutually learning ecosystem formed by Academia (A), Health System (H) and Community (C) in a team science based approach. The **LHS Model 2** (Ioachimescu O.C.) presents three major adaptations: (1) Community (C) becomes the center of the cycle, being involved in system co-design, in all operational leadership and governance, and part of the feedback loops of all 3 implementation arc segments; (2) PDCA improvement cycles are replaced by any improvement framework or approach the stakeholders choose; (3) domain-specific external evidence is incorporated at each ‘joint’. For example, innovations related to different areas (data science, dissemination and implementation, clinical practice, research and education) may be new business models (e.g., telemedicine clinical pathway, virtual classrooms, distributed clinical trials, etc.), novel methodological or technological approaches (e.g., use of artificial intelligence in data science and analytics, omics analytics, competence-based assessments, use of simulations in education and/or research, etc.), or externally validated new approaches (e.g., a new implementation framework, a more comprehensive theory, a new causal inference method, a new alliance or consortium, a new paradigm for pragmatic clinical trials, etc.)

As described by Friedman et al. [5, 6] the LHS is based on multidisciplinary exchange and collaboration, taking a systems approach in three distinct phases of the LHS cycle or *implementation arc* (Fig. 1 – model 0):

- Practice to Data (P2D): capturing real-world practice, results and outcomes as structured data. This phase ensures that the impact of implemented changes is systematically captured in practice, while comparing downstream outcomes with external benchmarks to detect variation, promote standardization, and spur innovation.
- Data to Knowledge (D2K): transforming data into actionable knowledge through analysis, synthesis, and integration of external evidence. During this phase, external evidence augments internal analyses and results to generate robust, generalizable knowledge.
- Knowledge to Practice (K2P): implementing new, computable knowledge into clinical practice, education, and research, and evaluating its impact. During K2P, knowledge is translated into action using implementation strategies that are informed by both internal pilots and external success stories or failures.

Friedman et al. [5, 6] add to their seminal framework two important modifications for effective LHS cycles: (1) that the first evolutionary step should be constituted by the formation of a learning community, and (2) that recycling only internal-generated evidence and good practices is often not sufficient, as the LHS cycle needs external evidence regularly curated, scrutinized and incorporated into the D2K phase (Fig. 1 – Model 0).

### Second movement (Largo): a modified LHS framework

We propose here to further modify the LHS cycle concept.

First, consistent with the CTSA hub model of a mutually learning ecosystem, we posit that Friedman’s learning community should be supported by three distinct and important pillars: medical school, health sciences university or academic enterprise [A], the health(care) system [H], and the community [C], constituting highly functional team science groups (or ‘ensembles’).

Second, we believe that P2D, D2K and K2P phases should have dedicated internal feedback or improvement loops, e.g., PDCA (Plan-Do-Check-Act) cycles (Fig. 1 - Model 1). The improvement loops for each phase are ‘framework agnostic’, as approaches may need to be tailored for different phases or activities.

Third, in our next theoretical iteration of the LHS cycle (Fig. 1 – Model 2), we:

1. emphasize the need to carefully design the structure and ensure optimal functionality of the three LHS cycle joints. The P2D, D2K and K2P phases are essential for the arc’s good functioning, as building capacity in data analytics, implementation science research and practice is of paramount importance. The transitions between phases are inherently mobile, hence represent structural and functional vulnerabilities, being prone to wear and tear, dysfunction or blockage. As such, at the organizational level, maintenance and function optimization of all three joints or connections become critical.
2. suggest that all three phases continuously incorporate not only practice-based data or new *internal evidence*, but also evidence-based practices as novel *external evidence* inputs. These relate to either new standards, breakthrough innovations or new methodological/business model approaches, and applied at each one of the junctures between phases.
3. recognize the importance of incorporating community stakeholders in all three phases. Consequently, community stakeholders become the center of the LHS cycle, providing support for P2D, D2K and K2P phases, not only part of the inception learning community that ‘plugs in’ within the P2D phase.

**Fig. 2:**
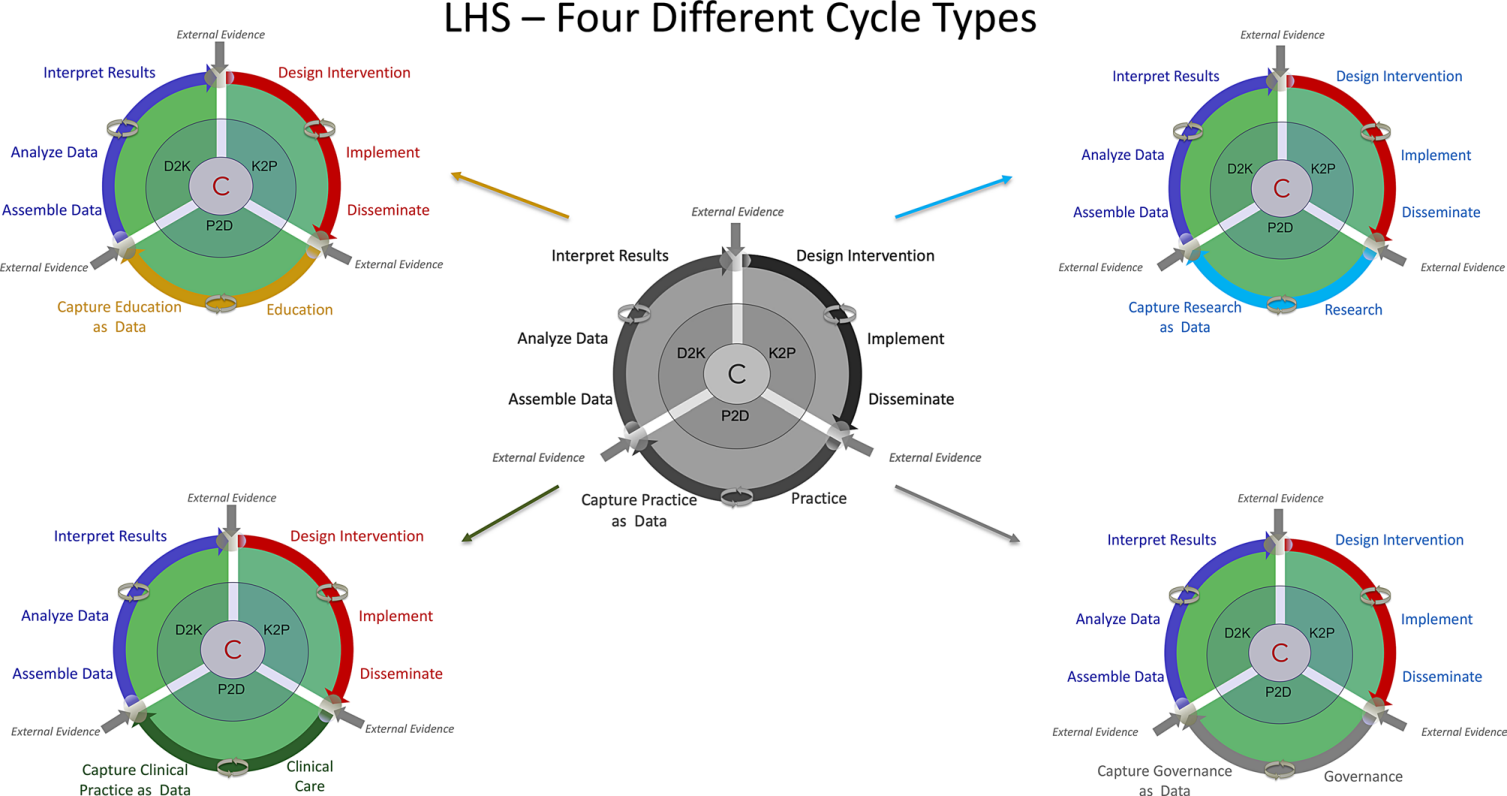
Four different types of LHS, based on ‘practice’ in clinical care, education, research, and governance activities. The term ‘practice’ in this paradigm is not restricted anymore to clinical care or praxis, but applies to all 4 domains (health care and promotion, research and discovery, education and training, leadership and governance)

In advanced health systems, the convergence of efficient clinical operations, breakthrough research activities, innovative educational efforts, and wise governance offers a unique opportunity to develop holistic or integrated LHS functionalities. We propose that LHS have distinct, mission-specific cycles for clinical, research, education, and governance, of various sizes and ‘cycling’ at different speeds. Each one of these four wheels includes the classic D2K, K2P and P2D phases, and ‘practice’ is represented by either clinical care, research operations, educational programs, or governance activities. At each junction, external evidence (published articles, professional practice parameters or guidelines, policy guidance, best practices, and benchmarking data) is systematically incorporated to contextualize internal insights, validate interpretations, and guide decision-making. At the heart of this model are community members and specific stakeholders, who actively inform priorities, co-design and co-create knowledge, and assess value across all three domains of clinical practice, research and education. Rather than passive recipients, stakeholders serve as navigators and co-designers of the learning cycles, ensuring alignment with real-world needs and values. This 4-wheeled, community-centered, and evidence-integrated LHS architecture (Fig. 2) supports dynamic learning and reciprocal improvement, enabling health(care) organizations to not only generate knowledge, but also to apply it swiftly, meaningfully and equitably across their missions.

The synergy of these four cycles enables LHS to address systemic challenges, such as activity fragmentation (e.g., dysfunctional clinical care) or slow knowledge translation, making research patient and health-centric, and education relevant for present and future workforce, governance deliberate in supporting these activities and amplifying their impact through transdisciplinary collaboration. Success depends on robust infrastructures for data analytics, stakeholder partnerships, and leadership commitment to institutionalizing learning cultures. By unifying these domains, advanced healthcare institutions can accelerate innovation, improve patient outcomes, and sustain transformative progress in healthcare delivery.

We believe that these LHS alterations represent key ingredients for lasting and highly effective LHS cycles in different domains or activities.

### Intermission (Intermissio): engine-drivetrain model - development methods

The CTSA hubs have complex structures, cores and functions, and one of their main goals is to facilitate workforce development, continuous learning and improvement at individual, institutional, and systems levels. Individuals (clinicians, scientists, investigators, staff, learners, community members, leaders, managers and administrators) provide structured education and training, facilitate mentoring, and help career development in clinical and translational science, emphasizing team science, leadership, community empowerment, and entrepreneurship. At institutional level, CTSA hubs create academic homes for translational research that coordinate cores, integrate data, build evaluation systems, streamline processes, and foster a culture of data-based, evidence-driven, interprofessional learning. At systems level, the national CTSA consortium acts as a test bed and dissemination pathway for new methods, best practices, tools, and policies, supporting LHS through co- or cross-hub learning, shared resources, and systemwide collaborative problem-solving.

The potential synergies between CTSA and LHS first appeared in the 2013 Institute of Medicine (IOM, now National Academy of Medicine) report named ‘CTSA Program at NIH: Opportunities for Advancing Clinical and Translational Research’ [7]. The reports makes a few important statements: “The CTSA Program does not exist in isolation; it is part of a larger clinical and translational research ecosystem that plays a vital role in an increasingly complex and dynamic U.S. health care system”, followed by “Across the United States, momentum is growing in support of a LHS in which researchers and health care providers design and implement care, evaluations, or research based on needs of specific communities and populations. The findings are disseminated to inform clinical practice and research models to improve health. A LHS is founded on the concept of continuous improvement and the imperative to translate *what we know* into *what we do*.” Then the report goes on to state that “such a system fuels greater value in health care by harnessing the promise of new technological capabilities, market opportunities, and policies. Thus, clinical and translational research is integral to a learning health care system” [7].

Before diving into specific frameworks for LHS and CTSA, it is important to clarify the terminology related to theories, models and frameworks, as used in implementation science and in the wider scientific literature [8]. Generally, a theory is a set of analytical principles or statements designed to structure the observation, its characterization and our understanding of the phenomenon. A good theory provides a clear theoretical explanation of how and why relevant relationships lead to specific events. A framework is a specific structure, overview, outline, system or plan not intended to provide explanatory relationships, but to be specific about which variables are important and the relationships between them. Frameworks tend to describe empirical phenomena by fitting them into a set of categories and subcategories. Lastly, a model is often a combination between a theory and a framework, and is a deliberate simplification of a phenomenon or a specific phenomenological aspect. Often times, models do not need to be completely accurate representations of the reality to have value [8].

Several theories (general, abstract constructs), frameworks (detailed, practical approaches), and few models (that integrate theories with frameworks for comprehensive grounding and simplified operationalization) have been proposed for both LHS and CTSA hubs, while implementation constructs dedicated to CTSA-LHS integration have not been developed yet. The existing constructs have either been inspired from each other’s phenotypic or endotypic characterizations (where the latter exist), or have evolved naturally towards similar infrastructures. To our knowledge, no unifying platform has been developed to date, in neither theoretical nor practical realms. This article stems from this identified gap, and based on a thematic overview of the published literature on the topic (see Table 1). An NCATS working group dedicated to the exploration of LHS-CTSA intersections [9] was assembled recently to systematically review some of the constructs on LHS and CTSA. The group recognized that different frameworks and approaches align in a continuum of definitions and choices, and that the focus should be specifically on LHS, trying to identify opportunities for CTSAs to intervene and facilitate the work of the learning organizations [9]. As such, the working group is looking to support partnerships between health care provision organizations and research support organizations [9]. However, due to the established role the CTSA hubs have in workforce development through education, training, and mentorship, we see opportunities beyond this approach, finding that the educational mission of the CTSA can serve as a unifying capability in LHS between clinical care and health promotion (on one side), and research, innovation and discovery (on the other side).

**Table 1:** A cursory overview of existing implementation constructs for LHS and for CTSA. Theories: general, theoretical constructs; Frameworks: detailed, practical schemas; Models: simplified combinations of theories and frameworks [8]

**Year**	**Model**	**Elements**	**Evaluation**
**LHS Frameworks**			
2016	LHS Science Competencies (AHRQ) [10]	Research questions & standards of evidence; Research methods; Ethics of research in health systems; Research management; Informatics; Engagement, leadership; Improvement; Implementation science; Ethics of implementation in health systems; Systems science; Health, healthcare equity and justice [framework]	Pro: a comprehensive aggregation of 38 LHS competencies from 8 distinct domains Con: even for the domains identified, a functional model is to be identified by the grant applicants
2021	LHS Consolidated Framework [11]	Five bodies of work (organizational learning, innovation and continuous improvement; Analysis of clinical data; Engagement of clinicians, patients and other stakeholders; building new knowledge and evidence, translating knowledge and evidence into improved practice) and 3 enabling conditions (invest LHS-dedicated resources; data systems and informatics infrastructure, employees with LHS expertise); supportive culture [framework-model(s)]	Pro: the 5 bodies of work reference elements from 10 + articles (‘with some synthesis’) that represent at times proposed functional models; the 3 enabling conditions represent a very nice aggregation for a ‘fundamental’ framework Con: lack of granularity for a unified, fully functional model of integration
2022	Adapted Interdisciplinary LHS Framework for Academic Health Centers [12]	Seven staggered structural components: organization and collaborations, performance, ethics and security, scientific approaches (including learning and research engines), data, information technology, and patient outcomes [theory-model]	Pro: one of the most functional, comprehensive and practical model published for LHS Con: pertains to academic LHS only; it may need more scripted frameworks for operationalization
2023	Academic LHS [13]	Six areas of emphasis: capitalizes on embedded academic expertise in health system sciences; engages the full spectrum of translational investigation from mechanistic basic sciences to population health; builds pipelines of experts in LHS sciences and clinicians with fluency in practicing in an LHS; applies core LHS principles to the development of curricula and clinical rotations for medical students, house staff, and other learners; disseminates knowledge more broadly to advance the evidence for clinical practice and health systems science methods; and addresses social determinants of health, creating community partnerships to mitigate disparities and improve health equity [framework]	Pro: differentiates activities by the three major domains (health care delivery, research and education); brings iterative improvement (cycles) into the construct Con: lacks evaluation matrix/framework; pertains to academic LHS only
2024	National Academy of Medicine Leadership Consortium (NAM) [14]	Foundational Elements (Science, Informatics, Incentives, Culture); Anchor features (Generate and use evidence through intelligent, rigorous learning architecture; Ensure that information systems are interoperable, secure, and accessible at the point of need; Reward improved outcomes, reduced costs, and deep engagement by both the workforce and the recipients of care; Promote and reinforce an openness to learning, inclusivity, and connection to the individuals and communities served); Shared Commitments (engaged, safe, effective, equitable, efficient, accessible, accountable, transparent, secure; adaptive) [theory-framework]	Pro: detailed, comprehensive, ex vivo dissection of foundational elements (major domains of the theory), anchor features (elements of the governance framework) and shared commitments (major elements of the functional trust principles) Con: practical applications of examples of success, with specific evaluative approaches
2024	LHS Maturity Model (Cope, E.) [15]	Pillars: Governance/Leadership; Socio-technical infrastructure; Improvement execution; Foundation: Culture and Values [theory]	Pro: simple, non-prescriptive (at granular level) arrangement of core competencies, which are currently undergoing validation; great approach to a LHS development assessment by maturity levels Con: lack of inherent elements of a functional model such as evaluative matrices
2025	Four Distinct Models of LHS (Friedman, C.P.) [16]	LHS types: participative. embedded researcher, pragmatic trials, programmatic [theory]	Pro: good phenotypic differentiation for various LHS types Con: descriptive, lacks frameworks for operationalization
2026	C^2^P^2^T^2^ Socio-technical and governance Architecture of LHS (Ioachimescu O.C.) [17]	Six elements: Climate, Culture, Policies, Processes, Talent and Technology [theory-framework]	Pro: enriches the LHS types per Friedman et al. by providing evaluation matrix Con: lacks validation
2026	LHS Intersections (Ioachimescu, O.C.) [18]	Arranging the translational spectrum domains (T0 to T5) as roads to a LHS roundabout, which regulates rules, rights, and permissions [theory]	Pro: a theory of governance for LHS Con: lacks implementation models, validation and evaluation frameworks
**CTSA Expertise and Frameworks**			
**Year**	**Model**	**Elements**	**Evaluation**
2019	Fundamental Characteristics of a Translational Scientist (Gilliland C.T.) [19]	Boundary Crosser; Domain Expert; Team Player; Process Innovator; Skilled Communicator; Systems Thinker; Rigorous Researcher [theory-framework]	Pro: good compilation of the general characteristics of the translational scientist Con: lack of contextualization for the environment and how these traits may be adapted in the ecosystem
2024	Translational Science Principles (NCATS) [20]	Prioritize Initiatives That Address Unmet Needs; Produce Generalizable Solutions for Common and Persistent Challenges; Emphasize Creativity and Innovation; Leverage Cross-Disciplinary Team Science; Enhance the Efficiency and Speed of Translational Research; Utilize Boundary-Crossing Partnerships; Use Bold and Rigorous Research Approaches [framework]	Pro: defines well the NCATS core 4 scientific and 3 operational principles of effective approaches to translational science Con: no universal model of integration between the framework and various theories
2024	Translational Science Engine (Ioachimescu, O.C.) [4]	CTSA hub-based team science approach with community in the center of activities [theory-model]	Pro: provides a strategic ‘zoomed-out’ view to modify the traditional linear translational science pipeline and to center team science around community engagement Con: it does not provide a descriptive script for implementation
2025	CTSA Hub Content Expertise (NCATS) [21]	Research across study lifecycle; Data science (Health informatics, clinical research informatics, bioinformatics); Community and stakeholder engagement; Continuous quality improvement; Dissemination and implementation within context of a learning health system; Solving roadblocks to clinical and translational research; Workforce development [framework]	Pro: aligns well with traditional NCATS elements and cores for CTSA hubs Con: lacks the ligands or ‘connective tissue’ between these elements (like any grant announcement, it invites submitters to find innovative ways to mesh these elements together)
2025	Translational science and related disciplines (Ioachimescu, O.C.) [3]	Translational Science, Implementation Science and related specialties [theory]	Pro: comprehensive view of intersections between translational science and 20 other disciplines Con: lacks granularity in capitalizing on ‘borrowed’ frameworks from related knowledge disciplines
2026	Innovation Orchards (Ioachimescu, O.C.) [22]	The role of CTSA hubs in ensuring vitality of ‘secular’ innovation trees, creating the novo ‘engineered’ innovation trees, or grafting operations for ‘hybrid’ innovation trees [theory-model]	Pro: a ‘zoomed-out’ view of integration of governance activities for ensuring success in innovation and scientific discovery; includes specific evaluation matrices; provides operationalization at individual (alpha), teams (micro), institution (meso) and systems (macro) levels Con: non-validated construct

### Third movement (Adagio): CTSA and LHS – the engine-drivetrain model

**Fig. 3:**
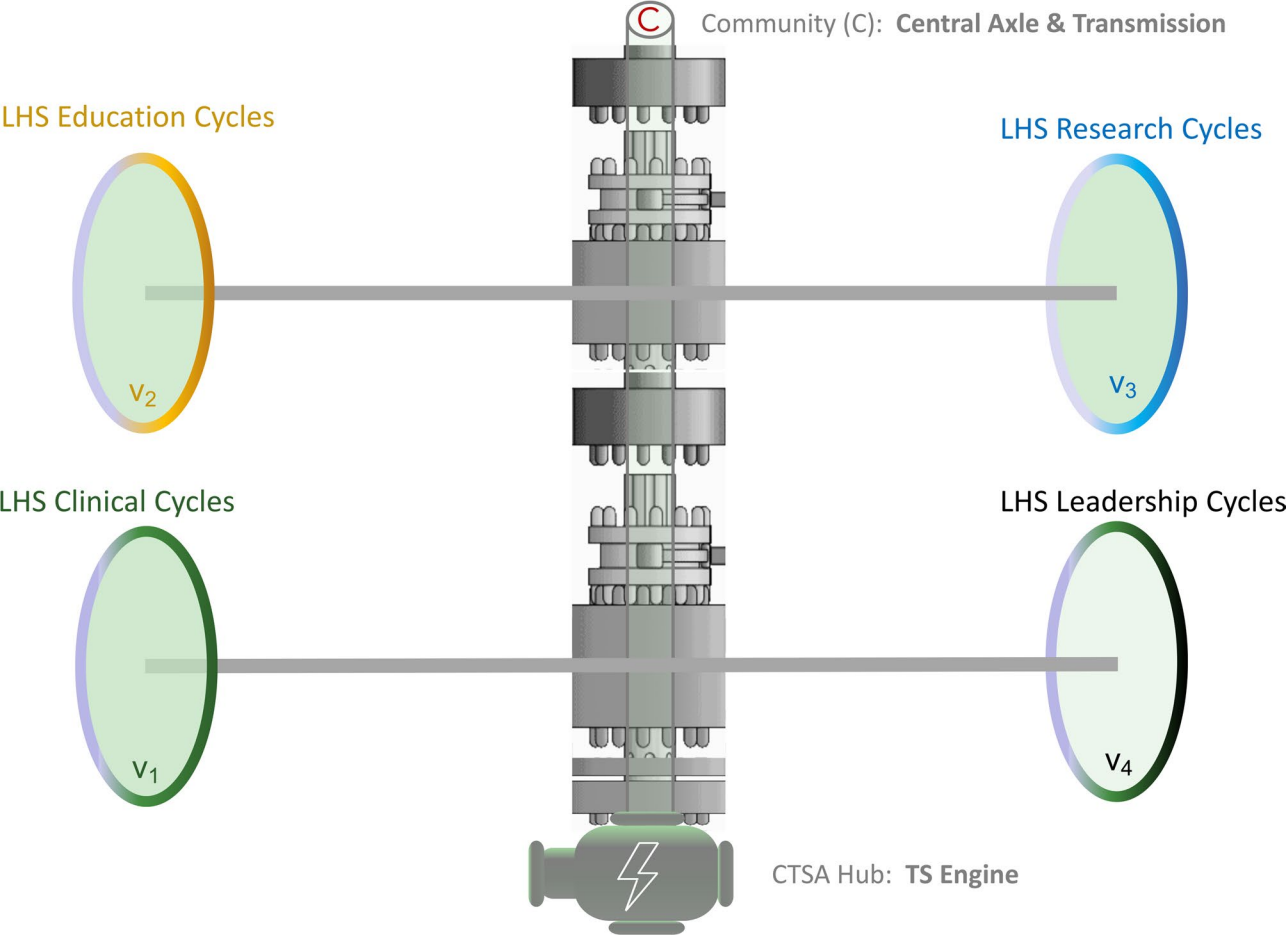
Engine-Transmission-Axle Machinery model of CTSA Hubs and Community partners mobilize the different types of LHS cycles. The CTSA hub serves as the LHS’ engine through its strategic partnerships with health systems and other institutions, via integrated data science and methodological infrastructures, leveraging the execution of the embedded and co-produced research, through its systems of measurement, dissemination and scaling, and by channeling its workforce development pipelines and the embedded culture of networked learning. The community partner is empowered as the mechanism’s axle, aligning the three missions (clinical care, research, and education) and acting as its transmission, ensuring their differential rotation at various, carefully tuned speeds (V_1_, V_2_, V_3_, V_4_), and functional vitality of the four LHS cycles

The proposed engine-drivetrain model (Fig. 3) operationalizes and optimizes the LHS framework, positioning CTSA as the organizational power source for continuous knowledge generation and application, with communities in the center, determining direction and ensuring that all cycles work at the right speeds and in concert [13, 23]. A CTSA hub can represent the central engine generating the cinematic energy for systematic knowledge creation, translation, and implementation across education, research, and clinical care domains, but also in governance, leadership and system administration. The engine’s capacity derives from and is augmented by its association with innovation-friendly clinical practices, from advanced educational capabilities, from the full spectrum of translational investigation spanning mechanistic basic sciences, clinical investigation, translational research, and population health, from the embedded academic expertise in health systems and translational science, from its established governance structures, its coalition convening capabilities, and its transdisciplinary collaborative capabilities [12, 13, 24–27].

In *clinical LHS cycles*, CTSA hubs power improvement by embedding internal evidence or research findings, data science and analytic capabilities, and robust evaluation processes into routine practice. These cycles integrate practitioners, researchers, and patients as active participants in an evidence-based continuum, ensuring that scientific discoveries reach the patients who need them most [12, 13, 25, 26, 28]. Community members, patients, caregivers, and local organizations enrich these cycles by defining meaningful outcomes and contextualizing care processes. Acting as the LHS transmission, community ensures that insights from clinical care are translated into changes that are culturally appropriate, trusted, and aligned with real-world needs.

In *research LHS cycles*, CTSA hubs can provide methodological rigor and capabilities, regulatory frameworks, investigative support and data science platforms that allow research to move efficiently and ethically. These cycles include the full T0-T5 translational science spectrum, producing evidence ultimately applicable to real-world settings [12, 13, 26, 27, 29]. The community, as the axle of this cycle, helps steer research direction by co-creating questions, participating as partners, and guiding dissemination. As LHS’ transmission, community partnerships carry research findings back into clinical settings and everyday life, accelerating or slowing down adoption (as needed), and optimizing impact.

Similarly, the *education LHS cycle*s can be energized by CTSA hubs through training, education and mentoring, preparing the future workforce represented by clinicians, investigators, staff, and community partners. These cycles transform academic expertise into workforce development and future capacity, preparing clinicians and investigators to participate in continuous evidence generation, quality improvement, dissemination and implementation [13, 24–27]. Community empowerment ensures that education remains grounded in lived experiences and population needs, while also anchoring values of trust, equity, good resource stewardship, and collaboration across systems.

Within the community-centered model, the *governance LHS cycle* functions as the integrative wheel that continuously aligns discovery, implementation, community priorities, and system accountability. At its core, governance establishes shared aims (equity, value, trust), sets data and ethical standards, and defines decision rights and permissions across academic (A), health system (H), and community (C) stakeholders. Through iterative, specific loops (P2D, D2K, K2P) leaders use real-time analytics, stakeholder input, and community partnerships to recalibrate priorities, allocate resources, and reduce structural friction. In this model, governance is not a static oversight body but a dynamic steering mechanism that converts CTSA-enabled translational capacity into equitable health impact, ensuring that learning, adaptation, investigation, improvement and accountability remain continuous rather than episodic.

*Community participation*,* involvement*,* engagement*,* and especially community empowerment* (CE) function as both the variable transmission mechanism that transfers power from the CTSA engine to each wheel, and the central axle around which all four cycles revolve. This dual role ensures that education, research, clinical care, and system governance remain aligned with community-defined priorities and health needs. Community partners participate not merely as research subjects but as active collaborators with synergy and trust, engaging in relationships characterized by collaboration and shared leadership [23, 30].

As LHS transmission, CE channels CTSA resources and expertise toward the most pressing health concerns, ensuring that training programs address real-world needs, research questions reflect community priorities, and clinical innovations reach populations experiencing health disparities. Community stakeholders shape research questions, participate in study design and conduct, and lead implementation of health advances of high importance to them [23, 24, 28, 30].

As LHS central axle, CE provides structural integration across all four cycles, ensuring that they work in concert rather than in isolation. This central positioning enables CTSA hubs to address social and economic determinants of health, create partnerships to mitigate disparities, and improve health equity across education, research, and clinical care missions. The unified taxonomy of health indicators that incorporates community priorities alongside professional metrics exemplifies this integrating function, providing common measures of success across all cycles [13, 23, 28, 30].

Communities do not merely receive innovation, but they actively manage and regulate speed, direction, and equity of its adoption. As such, CTSA hubs must be deliberate in trying to ensure that this transmission does not systematically disadvantage any segments of the population. First, CTSA hubs should invest in technical outreach and capacity-building, e.g., providing implementation support, data infrastructure, and practice facilitation to safety net clinics, rural practices, and community-based organizations that otherwise lack the resources to adopt new evidence fast and effectively. Second, hubs should emphasize the bidirectional nature of the CE and services’ co-design, working with community partners to adapt interventions to local context, culture, and constraints rather than assuming one-size-fits-all solutions. Third, CTSA hubs should use equity-focused metrics and implementation science frameworks to monitor who benefits, how quickly, and at what cost, and then adjust strategies accordingly. This way, CTSA hubs act as intentional ‘gear shifting’ mechanisms, reducing friction where inequities slow down adoption, and ensuring that learning and scientific discoveries propagate fairly across the entire system.

The four LHS cycles (clinical care and health promotion; research, innovation, and discovery; education and training, and good stewardship of the system) have certain synergies between them, also facilitated by the strength of the CTSA engine and by the robust community axle and its (variable) transmission. For example, an institution may calibrate a priori that one wheel has be developed to a specific size or scale, and to continue to move at a specific speed (V_1_, V_2_, V_3_ or V_4_ – Fig. 3). Then, some of the improvement cycles in one LHS type (e.g., in research) may accelerate its development and its revolution so much that (1) the CTSA hub engine may need to add extra torque or capabilities, e.g., to launch swiftly intramural funding mechanisms to help team science in a particular research area; to create disease-specific pilot awards; to dedicate a funding cycle to translational science capabilities; to contract with external experts in a contiguous field such as systems engineering to help the local implementation scientists in various projects, etc., or (2) the fast acceleration in the research LHS may demand that the education-based LHS creates specific curricula, micro-credentials, certificate, masters’ or other programs to keep up with the workforce development requirements in one specific area (e.g., data engineers, generative artificial intelligence [AI] specialists, prompt engineering capabilities, etc.). In another example, the advance of virtual clinical care may create the need for the education-focused LHS to create new professional skills and capacities (e.g., virtual or digital telehealth assistants, etc.), and perhaps cloud-based computing capacities may need to be augmented, etc.

The engine-drivetrain model facilitates bidirectional flow of data, information and knowledge across the translational science continuum. The national CTSA consortium enables networked, distributive learning across institutions, allowing multiple engines to work in parallel while maintaining community-centered direction [25, 27].

### Fourth movement (Moderato): examples of CTSA hubs in action in LHS

Consider an academic medical center seeking to reduce hospital readmissions for heart failure. As part of the LHS clinical cycle, the P2D segment is staffed by clinicians, patients, family members, and community partners, who together identify readmissions as a healthcare priority. Data on readmission rates, patient demographics, and care processes are collected, helped by national benchmarking data. The D2K segment is represented by data analysts and stakeholders, who review the literature on effective interventions, analyze local data, and identify gaps. Community members help interpret findings, highlighting barriers such as healthcare and medication access. During the K2P phase, a multidisciplinary team co-designs an intervention (e.g., enhanced discharge planning and follow-up), informed by external evidence and with inputs from community members. The intervention is piloted, with ongoing feedback from patients and staff. Outcomes are evaluated, and successful strategies are disseminated locally and externally. This clinical cycle is mirrored in the LHS research cycle (e.g., studying the intervention’s effectiveness) and in the LHS educational cycle through training and education for clinicians, staff and trainees in new protocols, with community members central to each process.

Over the years, CTSA hubs have supported multiple LHS-relevant innovations by leveraging their infrastructure to bridge research and practice, often with a strong focus on community engagement and data-driven improvement. Several community engagement models have been successfully implemented to increase minority participation in clinical trials. A cluster randomized trial using a church-based educational program showed statistically significant increases in participants’ interest to obtain clinical trial information at three and six months, though not in intention to join trials [31]. Another university-community partnership using a culturally tailored education program increased verified enrollment in a clinical trial registry among African Americans [32]. The COVID-19 Health Ambassador Program in South Los Angeles recruited health ambassadors from 17 faith-based organizations who led educational meetings and follow-ups for 152 African American older adults, successfully addressing vaccine hesitancy and chronic disease management [33]. Similarly, the Bridging Research, Accurate Information and Dialogue model created dialogue between community messengers and scientists to build trust in clinical trials [34]. Culturally-appropriate cancer clinical trial education programs have been developed for African American and Latino communities, showing significant increases in knowledge, trust in medical researchers, and willingness to participate in clinical trials [35, 36].

At the University of Wisconsin in Madison, investigators developed a convolutional neural network to function as a clinical decision support tool. The network analyzed in real time clinical notes to identify hospitalized adults at risk for opioid use disorder [37]. The model enabled continuous learning from clinical data by using natural language processing to map the first 24 h of electronic health record notes to standardized medical vocabulary, which was then fed into the deep learning model. Then the system identified at-risk patients and recommended consultations with addiction medicine specialists. The study showed clinical effectiveness, as the screener performed similarly to usual care in identifying patients for addiction medicine consultations; more importantly, the AI screener was associated with a 53% reduction in 30-day readmissions, with a marginal cost saving of $6,801 per readmission avoided [37].

Researchers supported by a CTSA developed a machine learning algorithm that estimated energy expenditure from commercial sensor data (accelerometer and gyroscope on a Fossil Sport smartwatch) and validated it in people with obesity [38]. The algorithm was tested in both laboratory and real-world conditions and addressed a critical gap, i.e., that the traditional actigraphy-based energy expenditure estimation is grossly inaccurate, especially in people with obesity [38]. The study demonstrated direct translation of research into a practical tool by showing that commercial wrist-worn devices can provide more inclusive and reliable energy expenditure measures [38]. This represents exactly the type of CTSA-supported innovation needed in LHS, creating an accessible, evidence-based tool that addresses health disparities and enables patient self-management through improved tracking of calories burned in populations with obesity.

The Veterans Administration (VA), which often partners with CTSA programs and which is trying to apply LHS principles in its operations, conducted a few successful projects addressing significant health priorities such as suicide prevention, opioid use and pain treatment, and access to virtual care. Those efforts used existing data to identify gaps in care and implement data-driven improvements to clinical practice. The VA Opioid Safety Initiative deployed multiple strategies, including education, pain management, risk mitigation, and addiction treatment, using electronic health records to identify high-risk patients and practitioners whose prescribing practices may not reflect best evidence. Since 2012, dispensed opioids decreased by 56%, and high-dose opioid prescriptions decreased by 77% [39]. In addition, the VA convened a dedicated state-of-the-art conference to develop research priorities for advancing opioid safety science and clinical practice [40].

Several CTSA hub programs shared core educational elements for spurring commercialization and entrepreneurship. The Entrepreneurship for Biomedicine program at Washington University included semester-long courses teaching core and advanced entrepreneurial skills, ethics, resilience, communication, and team-building, with mentorship from experienced entrepreneurs and opportunities for pitch presentations [41]. The THRIVE Fellowship program was another model where multidisciplinary teams followed biodesign principles through phases of healthcare innovation introduction, team formation with mentor selection and customer discovery, solution prototyping, and business plan development [42]. CTSA hubs broadly support commercialization pathways through technology transfer infrastructures, although the specific mechanisms vary institution by institution [43]. As CTSA program was designed to foster public-private partnerships and develop methods to ensure therapies reach patients who need them most [25], research examining CTSA effects on regional biomedical entrepreneurship found that these programs increased biomedical patents and entrepreneurship as measured by NIH Small Business Innovation Research grants, though the effect sizes were relatively small [44].

Multiple CTSA hubs developed electronic data warehouses to enable investigators to access and to analyze clinical data for research, continuous learning, dissemination and implementation, or quality improvement projects [45]. These systems support the methodical process of generating evidence from clinical practice, a core LHS function. Furthermore, the Trial Innovation Network, established by the CTSA consortium, has integrated more than 60 CTSA hubs into a functional network that reduced barriers to multicenter randomized clinical trials through novel tools, streamlined processes, resource optimization, and rapid communication pathways [46]. This infrastructure proved to be critically important during public health emergencies, including the COVID-19 pandemic and opioid crisis [46].

The University of California at Los Angeles (UCLA) CTSA hub’s interorganizational collaborations with the local county illustrated how CTSA hubs can partner with public health departments to address health concerns in low-income and at-risk populations [28]. These partnerships produced measurable impact across clinical, community, economic, and policy domains, directly addressing the mission to ‘impact the greatest health needs of Los Angeles and the nation’ [28]. Similarly, the Clinical and Translational Science Collaborative of Northern Ohio documented improvements in healthcare access, health outcomes, policy development, and economic benefits spanning local, national, and global contexts [47].

Recently, the use of generative artificial intelligence (AI) has helped advance fast many LHS functions, from interpretation of data and augmenting decision-making (data to insights), to supporting actionable outputs and reducing operational burdens (insights to practice), to enabling continuous feedback and tightening learning loops (practice to new data) [48–50]. For example, Large Language Models (LLMs) are being increasingly used to draft clinic and after-visit notes, visit summaries, replies to patient portal messages, and payor decision appeal letters, which clinicians can further review and edit [48]. Early studies show reductions in mental workload and burnout, creating feedback loops where note quality, safety issues, and user edits are monitored to iteratively improve prompts, guardrails, and workflows. While for some applications the CTSA hubs may be in catch-up mode, generative AI has clearly showed promise in clinical decision support and knowledge summarization, in codifying electronic health record unstructured data, in automated Quality Improvement (QI) reporting, in community engagement and stakeholder communications, in clinical trial matching and recruitment, sometimes in clinical trial protocol or guideline drafting, other times using synthetic data for save continuous model development, etc. To give a research example, the Generative Tensorial Reinforcement Learning model has been shown to be particularly adept at designing drugs tailored to specific biological mechanisms, ensuring that drugs interact optimally with biological targets relevant to an individual patient’s condition, towards personalized therapeutic plans [51]. Additionally, generative AI can help with pharmacogenomic optimization by analyzing data to predict individual therapeutic responses, leading to customized prescriptions and improved treatment outcomes [52, 53].

CTSA hubs also provide platforms for comparative effectiveness research by coordinating study design expertise, informatics, regulatory support, and community engagement programs [29]. The integration of dissemination and implementation science methods within CTSA-supported LHS initiatives addresses critical challenges including sustainability, generalizability, and health equity, areas often underrepresented in traditional LHS approaches [54]. Multiple CTSA training programs have developed pipelines of investigators fluent in translational science, implementation science, and health systems research, all competencies essential for operating within LHS [24, 25, 27]. These programs train researchers to work in transdisciplinary teams and engage communities as active participants in research design and implementation [24].

## Coda (Vivace): Conclusion

A modern health(care) enterprise is one in which health promotion and clinical operations; research, discovery and innovation; educational and training programs; and good governance efforts to prepare the workforce of the future are intermixed in various proportions. A LHS may provide the ideal environment, albeit complex and matrixed, for health centers to rapidly adopt evidence-informed or -based knowledge, and to translate research and innovation (from internal and external sources) swiftly into clinical praxis and governance. Clinical, educational, and research cycles align well with the general, external-looking missions of any health system. The explicit governance wheel, while not a traditional outside-looking mission, is likely an important in-wards design and strategic element, recognizing that scale and sustainability depend on the specific climate, culture, policies, procedures, and incentives. For a LHS to achieve its potential, organized and contextually relevant frameworks are needed to support implementation, reduce strategic, tactical or operational confusion, to analyze and calibrate quickly, and to develop effective and lasting transdisciplinary collaborations. So far, few LHS have done so.

Optimizing clinical operations to support research and education in academic settings requires a holistic, integrated approach, grounded in the LHS framework. By overlaying the learning cycles of clinical operations, research, education and governance, placing community stakeholders at the center of each type of cycle, and incorporating external evidence at each phase transition, LHS can create a dynamic, responsive, and equitable system of continuous improvement. This approach not only accelerates innovation and knowledge translation but also ensures that learning is meaningful, sustainable, and aligned with the needs and values of the organization and of the communities served.

## Data Availability

Not applicable.

## Abbreviations

A: Academia
C: Community
H: Health(care) system
AI: Artificial Intelligence
ANN: Artificial Neural Networks
CE: Community Empowerment
CTI: Community Transmission Index
CTSA: Clinical and Translational Science Award
LHS: Learning Health System
E: Expense (cost)
IOM: Institute of Medicine
IRB: Institutional Review Board
KPI: Key Performance Indicator
LLM: Large Language Models
MOU: Memorandum of Understanding
NCATS: National Center for Advancing Translational Science
NIH: National Institutes of Health
N5th & N95th: 5th & 95th national percentiles of global LHS translational velocities
PDCA: Plan-Do-Check-Act cycle
PDCA_d_: PDCA for the data analysis phase
PDCA_i_: PDCA for the implementation phase
PDCA_p_: PDCA for the practice phase
P2D: Practice to Data
D2K: Data to Knowledge
K2P: Knowledge to Practice
QI: Quality Improvement
Q_i_5th & Q_i_95th: 5th & 95th institutional percentiles of project speeds in LHS of i type
R: Revenue
ROI: Return on Investment
TS: Translational Science
UCLA: University of California Los Angeles
V: Translational Velocity (speed)
